# A Strange Story from Chicago

**Published:** 1887-04

**Authors:** 


### A STRANGE STORY FROM CHICAGO.

The fact that an inquest is to be held over the body of the victim in the second tragedy connected with the Red Bridge mystery, and that the peculiar circumstances still form as great a puzzle as they have for weeks, has led the police and others connected with the hypnotism, or the involuntary influence of one mind over another, forms an important element in the case. On March 4th, Frederick Wirth disappeared. A few days later Jacob Kuebler was arrested on suspicion of having murdered him, as Wirth was last seen in his company. From thence on until March 20th he was either in jail or out on bail and under the eyes of the police. On the evening of March 20th he entered the Twenty-second Street station, and speaking like a man in a trance, told the officers that the body of the murdered man (presumed to be Wirth) was in Ogden Slip held down under the piles. He was still talking when a stranger walked in and took him away, the officers making no protest, as they considered Kuebler crazy, and knew he was out on bail. The next day detectives on the case searched the Ogden Slip, and in the exact spot designated by Kuebler, pulled up the body of Peter Hansen. Hansen had disappeared on March 9th.

The theory is that the hypnotic influence of the real murderer, perhaps an acquaintance of Kuebler, was so strong that Kuebler was actively compelled, without the knowledge of the real murderer, to go to the station and tell what he did. The only explanation he has offered to his conduct is that he was eating supper when he saw a vision on the tablecloth of the body in the bottom of Ogden Slip. He supposed it was the body of Wirth, and went and told the officers at once. He still maintains that he has a feeling that Wirth's body is in Ogden Slip, and yet he cannot tell what makes him think so. He denies all knowledge of the death of Hansen and Wirth, however, and yet seems to wish to talk of them at intervals. Shortly after being first arrested Kuebler acted in so strange a manner that the police sent him to the insane department of the jail. Dr. Bluthardt examined him and pronounced him sane, although troubled with some strange nervous derangement. Bishop, the mind-reader, when told the circumstances, declared his belief in the probability that Kucbler was under hypnotic influence. Mr. Bishop expressed willingness to have Kuebler brought before him, and confidence that after a test he could throw some light on the mystery.—*N. Y. Tribune.*

